# Characterization of 3D Printed Yttria-Stabilized Zirconia Parts for Use in Prostheses

**DOI:** 10.3390/nano11112942

**Published:** 2021-11-03

**Authors:** Irene Buj-Corral, Daniel Vidal, Aitor Tejo-Otero, José Antonio Padilla, Elena Xuriguera, Felip Fenollosa-Artés

**Affiliations:** 1 Department of Mechanical Engineering, School of Engineering of Barcelona (ETSEIB), Universitat Politècnica de Catalunya, Av. Diagonal, 647, 08028 Barcelona, Spain; daniel.vidal@upc.edu (D.V.); ffenollosa@cimupc.org (F.F.-A.); 2 Centre CIM, Universitat Politècnica de Catalunya (CIM UPC), Carrer de Llorens i Artigas, 12, 08028 Barcelona, Spain; atejo@cimupc.org; 3 Department of Materials Science and Physical Chemistry, Universitat de Barcelona, Carrer Martí i Franquès 1, 08028 Barcelona, Spain; japadilla@ub.edu (J.A.P.); xuriguera@ub.edu (E.X.)

**Keywords:** zirconia, additive manufacturing, 3D printing, material extrusion, shrinkage, Sa, prostheses, porosity, mechanical strength

## Abstract

The main aim of the present paper is to study and analyze surface roughness, shrinkage, porosity, and mechanical strength of dense yttria-stabilized zirconia (YSZ) samples obtained by means of the extrusion printing technique. In the experiments, both print speed and layer height were varied, according to a 2^2^ factorial design. Cuboid samples were defined, and three replicates were obtained for each experiment. After sintering, the shrinkage percentage was calculated in width and in height. Areal surface roughness, S_a_, was measured on the lateral walls of the cuboids, and total porosity was determined by means of weight measurement. The compressive strength of the samples was determined. The lowest S_a_ value of 9.4 μm was obtained with low layer height and high print speed. Shrinkage percentage values ranged between 19% and 28%, and porosity values between 12% and 24%, depending on the printing conditions. Lowest porosity values correspond to low layer height and low print speed. The same conditions allow obtaining the highest average compressive strength value of 176 MPa, although high variability was observed. For this reason, further research will be carried out about mechanical strength of ceramic 3D printed samples. The results of this work will help choose appropriate printing conditions extrusion processes for ceramics.

## 1. Introduction

Zirconium dioxide, commonly known as zirconia (ZrO_2_) is a ceramic biomaterial that was first identified in 1789 [1]. However, it was not until 1969 that Helmer and Driskell [2] published the first research study about its use in biomedical applications: prostheses, dentistry, scaffolds, etc. In 1972, Garvie and Nicholson found that alloying zirconia with oxides such as calcia (CaO), yttria (Y_2_O_3_), and magnesia (MgO) could stabilize the tetragonal phase of zirconia, preventing its transition from the tetragonal to the monoclinic phase and producing ceramics with previously unseen crack resistance [3]. Specifically, nowadays yttria-stabilized zirconia (YSZ) is used to manufacture different kinds of prostheses such as dental, knee, or hip prostheses [4].

As an example, total hip replacement (THR) surgery, also known as hip arthroplasty (THA), needs to be carried out when a patient suffers a hip problem due to a variety of different conditions such as osteoarthritis, osteonecrosis, developmental dysplasia of the hip, and femoral neck fracture. The operation can relieve pain, restore function, and consequently, improve life quality in a significant manner [5]. Every year, more than 600,000 people undergo hip replacement surgery. Moreover, this number is expected to rise in the near future; a million operations are estimated to be performed by 2030 [6]. Therefore, it is important to carry out research in this area to improve the life expectancy of hip prostheses.

In prostheses, one option is to use materials that have a similar chemical composition to that of the bone and a similar structure as well as high biocompatibility. Calcium phosphate-based ceramics such as hydroxyapatite (HA) are an option. However, the biodegradation rate of HA is rapid and does not match the osteogenesis speed of bone [7] and its mechanical properties are not the most appropriate for hip prostheses [8]. In this way, its use is limited and, therefore, there are other options among the existing ceramic materials; one of them is yttria-stabilized zirconia (YSZ).

Zirconia offers several advantages over other materials used in the medical field: (1) significant reduction in the wear rate of implants [9]; (2) excellent biocompatibility [10]; (3) reduction in the risk of toxicity [11]; (4) preclusion of the possibility of corrosion resistance [12]; (5) appropriate mechanical properties for the manufacture of medical devices [10] (compressive strength of around 2000 MPa, bending strength of 900–1200 MPa [1], and fracture toughness of 5–10 MPa·m^1/2^ [13]). Additionally, it meets the requirements of ISO 13356 [14] regarding ceramic implants for surgery. However, the machining of zirconia is difficult due to its extreme hardness. Moreover, the material is brittle before the sintering operation and the thermal stresses due to the cutting operation may lead to breakage of the pieces.

Additive manufacturing (AM) represents an alternative that allows for obtaining more complex shapes and customized parts with porous structures if necessary [15]. There are seven categories of AM technologies according to ISO/ASTM 52900 [16]: (1) vat photopolymerization, (2) material extrusion, (3) material jetting, (4) binder jetting, (5) powder bed fusion, (6) direct energy deposition (DED), and (7) sheet lamination. Of all the AM technologies, one of the most common techniques used is the extrusion of a slurry-based material through a nozzle by applying a specific pressure. It has several variants. As an example, the use of a pyroelectric field allows fibers to be drawn on the top of a substrate [17].

Regarding the requirements of the prostheses, some areas need to be porous in order to help cell growth and ensure fixation by means of osseointegration [18]. On the contrary, other areas have to be compact, with low porosity in order to favor mechanical strength and with smooth surfaces that reduce friction and wear. It is also important to control the dimensions of the prostheses, which will be customized for each patient. The final dimensions of the parts will vary depending on the shrinkage coefficient after sintering. Thus, the present paper addresses the shrinkage, surface roughness, and porosity of zirconia compact parts manufactured by means of extrusion processes.

As for shrinkage of sintered YSZ samples, Muccillo et al. [19] reported values around 25%, and Li et al. [20] obtained values of 20.2%, 20.0%, and 21.6% in width, length, and height respectively. Both studies employed the usual sintering temperatures of, or close to, 1500 °C. On the contrary, Yu et al. [21] found higher shrinkage values of up to 36.8% using higher sintering temperature of 1700 °C and a sudden decrease to 1550 °C, in order to improve the density of the parts. On the other hand, a slight improvement in surface finish has been reported after sintering [22].

In extrusion printing methods, surface roughness in the lateral walls of the specimens depends on different printing conditions. For example, in porous fused deposition modelling porous (FDM) printed zirconia samples obtained with a linear structure and a raster angle of 45°, Buj et al. [23] found lowest roughness values around 23 µm. Shao et al. reported roughness values of 8.25 µm for sintered zirconia parts obtained with the 3D-gel printing technique [22]. On the other hand, Yu et al. [21] observed the shape of the stacked layers in YSZ samples in the build direction.

Porosity is defined as the percentage of void space in a solid [24]. In the solid parts of the prostheses, porosity should be as low as possible. It is well known that porosity affects the mechanical properties of the material: in general, the higher the porosity, the lower the mechanical strength of a part [25]. In that respect, Yu et al. [21] reported low porosity values between 1.719% and 3.961%. In another study [26], the porosity of sintered alumina substrate was found to be ~18.05 ± 1%. Peng et al. obtained dense YSZ structures with more than 94% of the theoretical density [27]. On the other hand, porosity might influence surface roughness [28]. For example, in pure titanium implants, lower porosity led to a decrease in surface roughness [29].

In terms of mechanical properties, Peng et al. [27] 3D printed fully and partially yttria-stabilized zirconia structures using the robocasting extrusion technology and showed flexural strength of up to 242.8 ± 11.4 MPa. Yu et al. [21] manufactured low porosity YSZ cylindrical samples with a diameter of 2 mm and a height of 4 mm, which showed a compressive strength of 1.56 GPa. Sakthiabirami et al. [30] developed hybrid porous yttria-stabilized zirconia scaffolds with a biopolymer (alginate/gelatine) in order to promote both excellent mechanical properties and bioactive performance. Zirconia scaffolds showed a strength of 68.2 MPa, which increased up to 89.8 MPa with the addition of the biopolymer. In another study [31], 8 mm diameter × 10 mm height parts were manufactured, in which YSZ was combined with PLA in different proportions. It was seen that the compressive strength range was below 1 MPa. Thus, this is a disadvantage when considering combining YSZ and PLA. Apart from extrusion, another AM technology that can be used for ceramics is DLP or direct light processing. Osman et al. [32] manufactured yttria-stabilized zirconia 3D printed parts at different angles with respect to the horizontal: 0°, 45°, and 90°. Specimens 3D printed at 0° showed a higher strength value (943.2 MPa) than 3D printed parts manufactured at 45° (822.3 ± 172 MPa) and 90° (834.4 ± 72 MPa). These values are similar to those of milled zirconia (800–1000 MPa) measured by Denry et al. [33].

The aim of the present study is to select appropriate extrusion 3D printing parameters for yttria-stabilized zirconia samples which can be used, for example, in ceramic prostheses. Low roughness is recommended in some areas of the prostheses that are in contact with other parts (although the 3D printed parts usually require a subsequent finishing operation after sintering). High dimensional accuracy is required if tailored prostheses are to be manufactured. As a general trend, the lower porosity, the higher mechanical strength is. This last property is necessary for the appropriate performance of the protheses during use. In the present work, design of experiments (DOE) was used with a two-level factorial design, in which print speed and layer height were defined as variables. The results will be helpful in order to manufacture ceramic parts by means of extrusion 3D printing techniques.

## 2. Materials and Methods

### 2.1. Zirconia Samples Preparation

The aqueous ceramic suspensions were prepared for 2 min at 2000 min^−1^ in a mixer with rotation and revolution movement under vacuum pressure reduction enabling simultaneous dispersion of materials and the elimination of air bubbles (ARV-310P Thinky Corporation, Tokyo, Japan). The ceramic powder, 3 mol% yttria-stabilized zirconia (HSY-3B, Daiichi Kigenso Kagaku Kogyo Co., Ltd., Hirabayashiminami, Japan), and dispersant (Dolapix PC75, Zschimmer & Schwarz, Lahnstein, Germany) were added to a Pluronic F-127 (Sigma-Aldrich, St. Louis, MO, USA) stock solution of 25 wt% to prepare the inks. The 0.5 wt% dispersant against the 3YSZ powder amount was added for a final solid load of 40 v%. The ceramic powder has a D50 value of 0.7–1.5 µm. It corresponds to the median of the particle size distribution.

### 2.2. 3D Printing and Sintering Process

**Figure 1:**
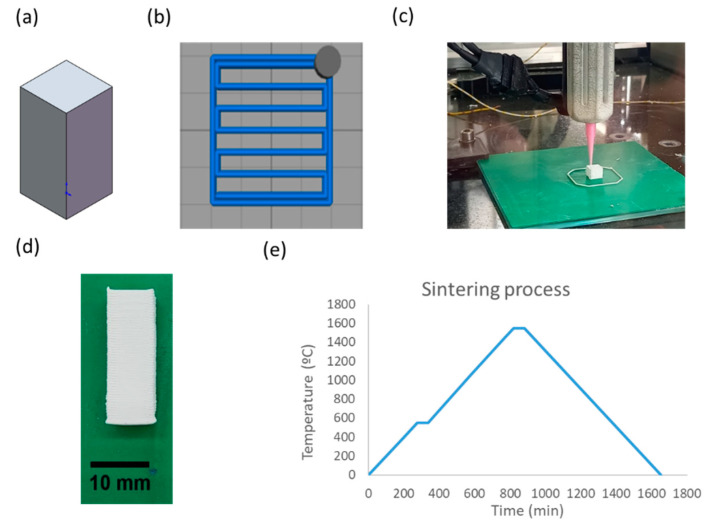
(**a**) Cuboid sample of 9 × 9 × 20 mm^3^; (**b**) example of a rectilinear infill pattern with a raster angle of 0°; (**c**) extruder; (**d**) example of a printed YSZ part using imaging software (ImageJ); (**e**) sintering cycle.

Cuboid samples of 9 × 9 × 20 mm^3^ were printed (Figure 1a) with a rectilinear infill pattern, with a raster angle of 0° in all layers (Figure 1b), without a shell or bottom or top walls. An AMFEED-PRINTER extruder (CIM-UPC, Barcelona, Spain) was used (Figure 1c). The material extrusion method was selected for printing the samples based on the extrusion of a liquid-phase ink through a nozzle with a controlled flow rate. Nozzle diameter was 0.58 mm, the infill was 99%, and the extrusion multiplier factor was 100% in all cases. The room temperature of the machine varied between 27 °C and 29 °C.

Table 1 defines the speed and layer height values used in the different experiments, according to a 2^2^ full factorial design, which was defined with Minitab^®^ 19.2 version (Minitab Inc., State College, PA, USA). Three replicates of each experiment were performed.

After printing, the samples were placed inside an oven at 37 °C for 24 h. An example of a printed part is presented in Figure 1d. Subsequently, the samples were sintered. The sintering cycle is presented in Figure 1e. The maximum sintering temperature was 1550 °C [34]. In order to minimize the presence of cracks, the sintering rate was 2 °C/min [35].

### 2.3. Areal Surface Roughness

Surface roughness was measured with Smartproof 5 confocal equipment (Zeiss, Oberkochen, Germany). A 20X magnification lens was employed. The use of optical equipment avoids mechanical contact between the device and the samples, preventing them from being damaged [36]. The lateral measurement uncertainty of the equipment is ±(0.1 µm + 0.008 × L), while the vertical measurement uncertainty is ±(0.1 µm + 0.012 × L).

**Figure 2:**
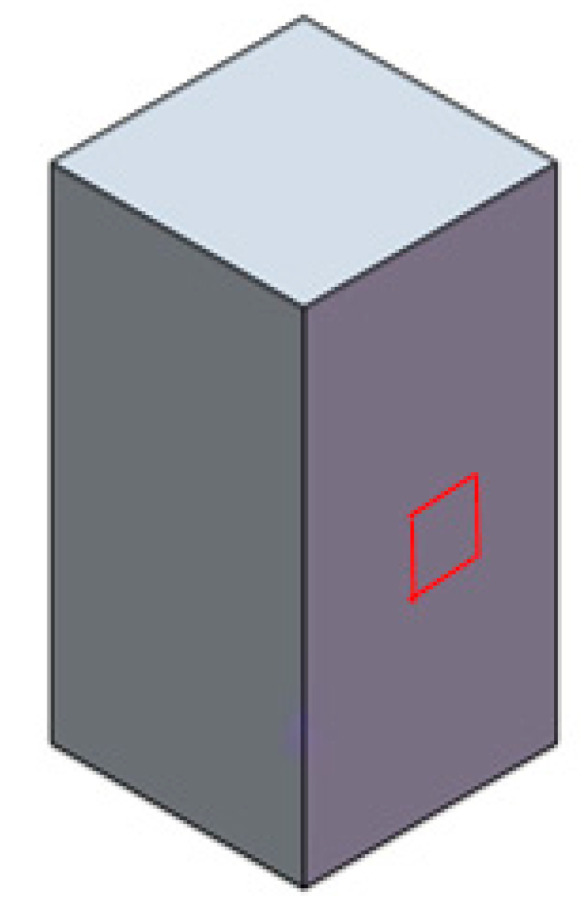
Schematic of the area where roughness was measured on the lateral wall of the cuboids.

Areal arithmetical mean Sa was considered, according to the ISO 25178 standard [37]. One measurement was performed on each sample, on one lateral wall of the cuboid (red area in Figure 2). The measured area was 0.5 mm × 0.5 mm (the picture is not to scale).

### 2.4. Shrinkage Measurement

A digital micrometer (Mitutoyo, Kawasaki, Japan) was used to determine the dimensions of the sides and the height of the cuboid samples after sintering [36]. Those dimensions were compared to the theoretical dimensions of the parts before sintering (9 × 9 × 20 mm^3^). For both the width and height of the specimens, shrinkage values were determined as percentages.

### 2.5. Total Porosity Measurement

The density of the samples was calculated by the determination of the weight and the dimensions of the samples. Weight was measured on a Kern 440-33N scale (Kern & Sohn GmbH, Balingen, Germany). Dimensions were measured with a digital micrometer (Mitutoyo, Kawasaki, Japan). Total porosity was calculated by a comparison of the density with the theoretical density of 5.68 g/cm^3^ for YSZ [38].

### 2.6. Mechanical Characterization

The compressive stress of the printed and sintered 3D cuboid samples was determined using an INSTRON 3366 universal testing machine (Instron, Norwood, MA, USA) with a load capacity of 10 kN at a cross-head speed of 0.13 mm/min. The samples were tested perpendicularly to the long axis under compression until crushed The maximum compressive stress attained during compression is defined as the force applied on the object divided by the area of the cross-section of the object. Three samples per group were tested.

### 2.7. Structural Analysis

The surface morphology of the 3D printed YSZ samples was evaluated by scanning electron microscopy (SEM), ESEM Quanta 200 FEI, XTE 325/D8395 (FEI, Hillsboro, OR, USA). The system was operated at 20 kV as accelerating voltage. The surface morphology analyses were performed by taking secondary electrons (SE) and backscattered electrons (BSE) images.

## 3. Results

### 3.1. Surface Roughness

The areal arithmetic roughness, S_a_, of the different experiments is shown in Table 2.

As expected, high roughness values correspond to experiments 2 and 4, printed with a layer height of 0.4 mm. Low roughness values correspond to experiments 1 and 3, with a low layer height of 0.2 mm. Roughness in the lateral walls of FFF printed parts depends greatly on layer height [39,40].

**Figure 3:**
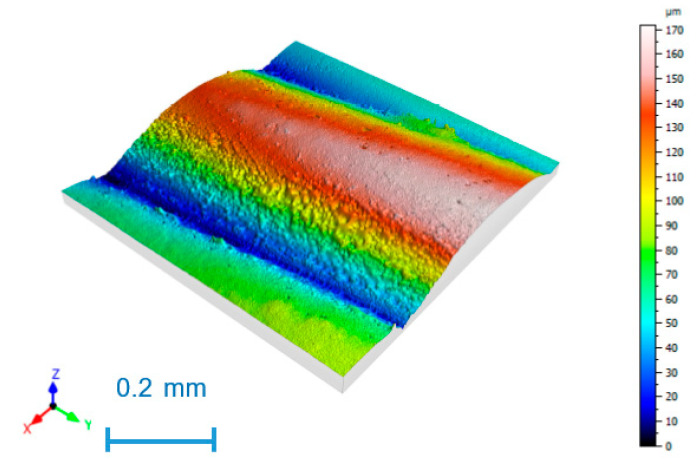
Surface topography for experiment 2.

**Figure 4:**
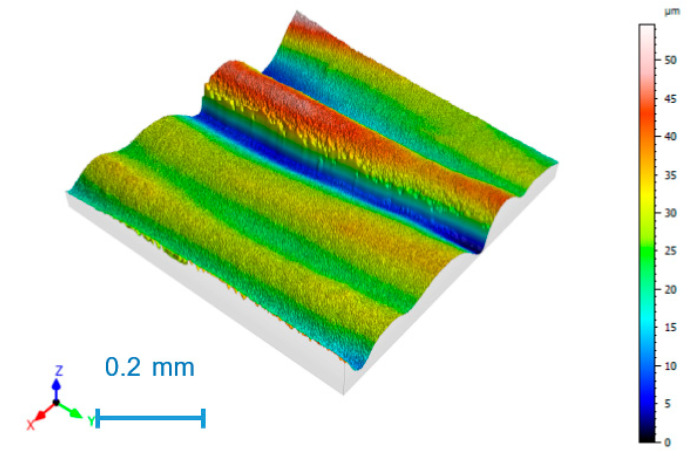
Surface topography for experiment 3.

As an example, Figure 3 shows the surface topography of experiment 2, with the highest S_a_ value; Figure 4 corresponds to experiment 3, with the lowest S_a_ value.

**Figure 5:**
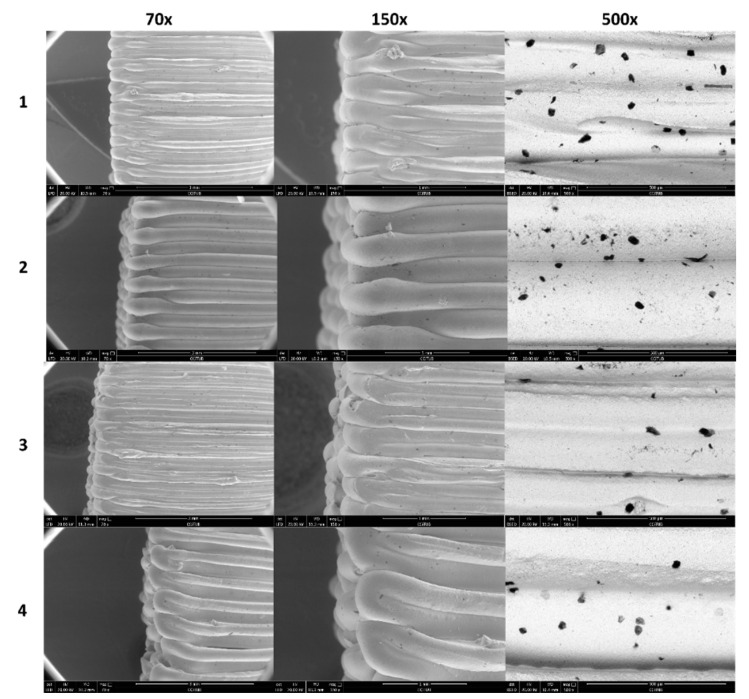
SEM images of the lateral wall of the cuboid of printed lines direction. SE images are 70X and 150X, and BSE images are 500X. Numbers 1, 2, 3, and 4 correspond to the different experiments defined in Table 1.

The highest roughness value with a mean S_a_ value of 24.6 µm is found in experiment 2 (Figure 4), corresponding to low speed and high layer height. The lowest roughness value, with a mean S_a_ value of 9.4 µm, was obtained in experiment 3 (Figure 5), with low layer height and high speed. Parallel printing layers were observed in all cases, with rounded peaks and sharp valleys, corresponding to the correct printing processes [23].

### 3.2. Shrinkage

Table 3 presents the results of the mean width, standard deviation of width, mean height, standard deviation of height, and shrinkage.

Experiments 1 and 2, obtained at low speed, showed lower shrinkage in height and higher shrinkage in width than experiments 3 and 4, obtained at high speed.

### 3.3. Density and Total Porosity

Table 4 shows the mean and standard deviation values of density and total porosity for the different experiments.

The experiments obtained at low speed (1 and 2) show the highest weight than the experiments obtained at high speed (3 and 4), suggesting that low speed helps to properly deposit the material.

### 3.4. Mechanical Strength

In the present work, the mechanical characterization was focused on the compressive strength of YSZ samples, which are summarized in Table 5. It was seen that the 3D printed samples with low print speed and low layer height resulted in having better mechanical properties.

The highest mean compressive strength of 172 MPa corresponds to experiment number 1, obtained with low speed and low layer height. It corresponds to the sample having the lowest porosity (Table 4), suggesting that, in this case, better densification was achieved. Experiment 2, obtained with low speed and high layer height shows a relatively high mean compressive strength value of 134 MPa. Thus, low speed favors higher compressive strength than high speed. However, experiments 1 and 3, corresponding to low layer height, show higher variability than experiments 2 and 4, obtained with high layer height. In addition, compressive strength values are lower than those of sintered low porosity YSZ samples [33], between 800 and 1000 MPa. Further research is required in order to reduce porosity of the printed samples, for example using higher sintering temperature. On the other hand, the use of a higher nozzle diameter would allow for using a higher ceramic load of the ink. However, higher nozzle diameter is usually related to higher layer height, and such conditions are likely to provide higher surface roughness.

### 3.5. Structural Analysis

**Figure 6:**
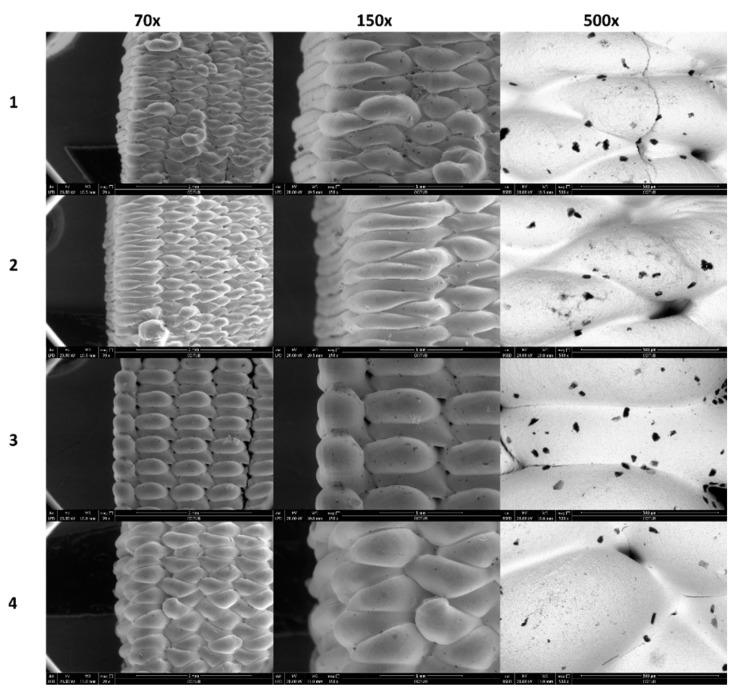
SEM images of the lateral wall of the cuboid of direction change of printed lines. SE images are 70X and 150X, and BSE images are 500X. Numbers 1, 2, 3, and 4 correspond to the different experiments defined in Table 1.

Figure 5 and Figure 6 show the surface morphology analysis of the YSZ 3D printed samples by SEM. Images corresponding to Figure 5 were made on the lateral wall of the cuboid, in which the entire printed line is observed. Instead, images corresponding to Figure 6 were made on the lateral wall of the cuboid where the change in the printing direction is shown.

In this way, it is possible to extract several conclusions. The most obvious is the influence of layer height (0.2 mm vs. 0.4 mm) of the 3D printed samples by looking into the 70X and 150X magnification. The lower the layer height, the lower the thickness of the final line is.

As can be seen in Figure 5, samples 1 and 2 have lines with constant thickness and fewer surface defects. In addition, samples 1 and 3 present higher overlap due to lower layer height.

Secondary electron (SE) images (70X and 150X) of Figure 5 and Figure 6 show some dark spots that correspond to pores, confirmed by backscattering electron (BSE) images (150X). The size and distribution of these surface pores seem to be independent of printing conditions.

## 4. Discussion

In the present paper, YSZ cuboids were printed by means of the extrusion method and subsequently sintered in order to achieve their final strength. Different properties of the samples were measured: shrinkage, surface roughness porosity, and compressive strength.

These printed ceramic parts could be used, for example, to manufacture prostheses. The ultimate purpose of the research is to print ceramic acetabula. In this sense, in a previous paper porous structures were obtained in ceramic [23]. In addition, scaffolds with a trabecular structure were obtained in plastic material by means of the fused filament fabrication (FFF), also known as the fused deposition modeling (FDM) technique [41]. The manufacture of acetabulum prostheses will be possible in the future with a compact hemispherical shape and a porous outer layer.

In the present work, the values of the printing parameters were selected in order to obtain a relatively good surface finish and dimensional error. For instance, a low nozzle diameter of 0.58 mm was selected with a layer height of either 0.2 mm or 0.4 mm. The use of such nozzle diameter, however, limits the ceramic load of the ink. For this reason, some porosity is found in the printing samples leading to lower mechanical strength than that of solid ceramic samples.

Roughness values of S_a_ = 9.4 µm were obtained in the lateral walls of the samples under certain printing conditions: low layer height and high speed. Although few papers address the influence of extrusion printing parameters on the roughness of ceramic parts, this topic has been addressed in the extrusion of plastic materials by means of a similar technique, fused deposition modelling (FDM). In this case, it is widely accepted that the lower the layer height, the lower the peaks of the profiles are and lower roughness values are obtained [42]. Regarding print speed, the use of high speed has been reported to reduce surface roughness [43], in a similar way as in the present work. On the contrary, other authors found that roughness increased with print speed [44]. On the other hand, similar roughness values to those obtained in the present paper were reported by Shao et al. [22] for zirconia, around 8 μm. In the field of implants, surfaces with S_a_ ≤ 1 µm are considered smooth, while those with S_a_ > 1 µm are described as rough [45]. Therefore, the samples in the present study are considered to be rough and would require a subsequent polishing operation if they were to be used, for example, in a joint between two prostheses.

Shrinkage values below 28% in width and below 24% in height were obtained for YSZ in this case, which are similar to those reported by Muccillo et al. [19], around 25%, and by Li et al. [20], below 22%. On the contrary, Yu et al. [21] found higher shrinkage values of up to 36.8% for YSZ samples that were sintered at a high temperature of 1700 °C.

In compact parts of the prostheses, the required porosity should be as low as possible, in order to reduce the presence of voids or cracks that would reduce their mechanical strength [46]. For example, Deng et al. reported the highest mechanical strength in zirconia samples with low porosity [47]. In the present work, the lowest mean porosity value of 12.36% was obtained with low speed and low layer height.

In porous YSZ samples of up to 90% porosity, obtained through the tertbutyl alcohol (TBA)-based gel-casting process, Dong et al. [48] reported low strength values between 0.34 and 0.66 MPa. If the porous YSZ is reinforced with 10% vol. Al_2_O_3_ fibers, the compressive strength increases up to 100.2 MPa. In densely printed samples, Yu et al. [21] reported higher compressive strength values of 1.56 GPa. However, in that case, zirconia pastes with a higher load (60 vol%) than that of the present work (40 vol%) was used. A nozzle diameter of 1 mm was also higher than the one used in the present research (0.58 mm). The higher the nozzle diameter, the higher the layer height is recommended to be selected, and this worsens the surface finish of the lateral walls of the parts. Thus, further research is required in order to improve the mechanical properties of the parts without compromising their surface finish. As mentioned in the results, in the present work, 3D printed samples with low print speed and low layer height resulted in having better mechanical properties, which is confirmed by Jaya Christiyan et al. [49], probably because such conditions lead to better bonding between layers. Such conditions will be used in future works with higher nozzle diameter, higher ceramic load, and/or higher sintering temperature, in order to reduce porosity and improve the mechanical strength of the printed parts.

## 5. Conclusions

The present paper presents results about areal roughness Sa, shrinkage, porosity, and compressive strength of printed and sintered YSZ samples. The main conclusions of the paper are as follows:

- Relatively low S_a_ values below 10 μm were obtained when combining low layer height and high speed. Layer height is the most important variable affecting roughness in this case.
- Shrinkage in height was higher for samples obtained with high speed, while shrinkage in width was higher for samples with low speed. In all cases, shrinkage values exceeded 19% as is usual in zirconia specimens.
- Total porosity ranged between 12.26% and 24.32%. The lowest mean porosity corresponds to the combination of low layer height and low speed. Layer height was the most influential variable on porosity in this case.
- Compressive strength is directly related to the porosity of the parts. Compressive strength values up to 172 MPa were obtained when low layer height and low speed were employed. Further tests are required, with the use of higher ceramic load and/or higher nozzle diameter. However, the last option would imply the use of higher layer height and thus the surface finish of the lateral would worsen.

The present work will help to select the appropriate printing conditions in extrusion methods, so as to obtain parts with relatively low roughness, low porosity, and high compressive strength to be used, for example, in prostheses.

## Figures and Tables

**Table 1:** Print speed and layer height values of the different experiments.

Experiment Number	Print Speed (mm/s)	Layer Height (mm)
1 (3–0.2)	3	0.2
2 (3–0.4)	3	0.4
3 (7–0.2)	7	0.2
4 (7–0.4)	7	0.4

**Table 2:** Mean and standard deviation (SD) of areal average roughness S_a_ of the printed samples.

Experiment	Mean S_a_ (μm)	SD S_a_ (μm)
1 (3–0.2)	12.4	1.7
2 (3–0.4)	24.6	3.5
3 (7–0.2)	9.4	2.7
4 (7–0.4)	20.2	2.9

**Table 3:** Mean and standard deviation (SD) of width, height, and shrinkage of the printed samples.

Experiment	Mean Width (mm)	SD Width (mm)	Mean Height (mm)	SD Height (mm)	Shrinkage in Width (%)	Shrinkage in Height (%)
1 (3–0.2)	6.49	0.20	15.99	0.42	27.99	20.05
2 (3–0.4)	6.70	0.41	16.03	0.37	25.57	19.88
3 (7–0.2)	6.96	0.41	15.24	0.56	22.70	23.80
4 (7–0.4)	6.90	0.43	15.55	0.21	23.31	22.28

**Table 4:** Density and total porosity of the printed samples. SD is the standard deviation.

Experiment	Mean Weight (g)	SD Weight (g)	Mean Density (g/cm^3^)	SD Density (g/cm^3^)	Mean Total Porosity (%)	SD Total Porosity (%)
1 (3–0.2)	3.35	0.13	4.98	0.05	12.36	0.90
2 (3–0.4)	3.35	0.08	4.68	0.31	17.68	5.52
3 (7–0.2)	3.21	0.43	4.25	0.38	18.84	6.77
4 (7–0.4)	3.08	0.16	4.30	0.10	24.32	1.72

**Table 5:** Compressive strength of the YSZ samples.

Experiment	Compressive Strength (MPa)	SD Compressive Strength (MPa)
1 (3–0.2)	172	86
2 (3–0.4)	134	19
3 (7–0.2)	74	55
4 (7–0.4)	60	14

## Data Availability

Not applicable.
